# Next-Generation Pathology Using Multiplexed Immunohistochemistry: Mapping Tissue Architecture at Single-Cell Level

**DOI:** 10.3389/fonc.2022.918900

**Published:** 2022-07-29

**Authors:** Francesca Maria Bosisio, Yannick Van Herck, Julie Messiaen, Maddalena Maria Bolognesi, Lukas Marcelis, Matthias Van Haele, Giorgio Cattoretti, Asier Antoranz, Frederik De Smet

**Affiliations:** ^1^ Translational Cell and Tissue Research Unit, Department of Imaging and Pathology, KU Leuven, Leuven, Belgium; ^2^ Department of Oncology, KU Leuven, Leuven, Belgium; ^3^ The Laboratory for Precision Cancer Medicine, Translational Cell and Tissue Research Unit, Department of Imaging and Pathology, KU Leuven, Leuven, Belgium; ^4^ Department of Pediatrics, University Hospitals Leuven, Leuven, Belgium; ^5^ Pathology, Department of Medicine and Surgery, Università di Milano-Bicocca, Monza, Italy; ^6^ Department of Pathology, Azienda Socio Sanitaria Territoriale (ASST) Monza, Ospedale San Gerardo, Monza, Italy

**Keywords:** multiplexed immunofluorescencence and immunohistochemistry, spatial profiling, single-cell ‘omics, tissue architecture analysis, methods for spatial profiling

## Abstract

Single-cell omics aim at charting the different types and properties of all cells in the human body in health and disease. Over the past years, myriads of cellular phenotypes have been defined by methods that mostly required cells to be dissociated and removed from their original microenvironment, thus destroying valuable information about their location and interactions. Growing insights, however, are showing that such information is crucial to understand complex disease states. For decades, pathologists have interpreted cells in the context of their tissue using low-plex antibody- and morphology-based methods. Novel technologies for multiplexed immunohistochemistry are now rendering it possible to perform extended single-cell expression profiling using dozens of protein markers in the spatial context of a single tissue section. The combination of these novel technologies with extended data analysis tools allows us now to study cell-cell interactions, define cellular sociology, and describe detailed aberrations in tissue architecture, as such gaining much deeper insights in disease states. In this review, we provide a comprehensive overview of the available technologies for multiplexed immunohistochemistry, their advantages and challenges. We also provide the principles on how to interpret high-dimensional data in a spatial context. Similar to the fact that no one can just “read” a genome, pathological assessments are in dire need of extended digital data repositories to bring diagnostics and tissue interpretation to the next level.

## Introduction

For centuries, medical sciences have tried to achieve a deep understanding of the human body, both in health and disease. Twenty years ago, a major hurdle was crossed with the mapping of the human genome (1). However, it is now becoming clear that one cannot just “read” a genome and subsequently understand or predict the principles that underlie human biology or disease. The sole true interpreters of the genome are cells, and understanding how the genome functions within cells, how cells form tissues and dynamically remodel their activities when they progress towards disease, is among the greatest scientific and technological challenges of our era. The goal is no longer to find differences in “bulk” genomic readouts but rather to see explicit changes in a specific set of cells and to predict their behavior. In this light, multiple human cell atlas initiatives are working towards describing every cell of the human body, as a reference map to accelerate progress in biomedical science (2–5). These ambitious projects – similar in scale to the human genome project – aim to chart the different types and molecular properties of all human cells in health and disease, for which a multitude of organ-oriented working groups are mapping the single-cell composition and their spatial architectures. Technological advances in the field of single-cell ‘omics’ such as single cell genomics, epigenomics, transcriptomics, and proteomics, and even their combinations in a multi-omic setting (6, 7) are now rendering it possible to map physiological features of each individual cell in an organ as a functional unit. However, current methods still mostly require that cells are dissociated and removed from their original microenvironment, thus destroying valuable information about their location and interactions – information that is crucial to understand many disease patho-physiologies. The next, ongoing step is aimed at describing single-cell features in their natural microenvironment (8).

At the moment, understanding cellular functions at single-cell level within the context of a tissue is primarily done by expression profiling, using either transcriptional (9) or protein-based multiplex methods in pathological tissue sections (10), even though the realm of spatial omics keeps on growing fast (11, 12). While methods for enhanced spatial omics are only now starting to become available, pathologists have been evaluating cells in tissue sections using classical antibody- and morphology-based (i.e. H&E staining) methods for decades (13). Daily clinical practice is mostly performed using classical (chromogenic) immunohistochemical (IHC) methods that allow the simultaneous assessment of one or two proteins in a single tissue slide, which are mostly evaluated in a visual, semi-quantitative way by a pathologist (14). Novel technologies and methods, that will be discussed in this review, are now making it possible to perform quantitative spatial, antibody-based expression profiling of dozens of protein markers in a single tissue section. This will, when carefully selected, provide deeper insights in disease states while offering the ability to study cell-cell interactions, precisely define disease-related niches, all within the original context of the tissue (see below).

Such technology also comes along with multiple challenges as well. First, a careful selection of the right technology to answer the biological question is crucial: as we will discuss below, this is defined by the type and size of a tissue, the number of samples that need to be processed, and the number of markers that need to be interrogated. Second, besides the technical hurdles that need to be overcome to collect high quality, pathology-grade images in which each marker is carefully monitored, storing and processing the large volumes of image data also pose a significant logistic and infrastructural challenge. Finally, the plethora of information that is obtained from these analyses is also becoming of such magnitude and complexity that mere eyeballing of a tissue by a pathologist or researcher is no longer sufficient to properly extract information and interpret expression patterns. Similar to the fact that mutations in a genome need to be interpreted for biological and clinical relevance, pathological assessments also need the installation of suitable analysis algorithms and extended digital data repositories to bring diagnostics and tissue interpretation to the next level.

In this review, we provide a comprehensive overview of the available technologies for multiplexed immunohistochemistry, their advantages and challenges, and provide the basic principles on how to interpret high-dimensional data in a spatial context.

## Methods for multiplexed immunohistochemistry

Lately, the armamentarium of technologies for antibody-based multiplexed IHC is rapidly growing. While several technologies have been described in literature performing manual IHC protocols, there is a clear trend of seemingly ‘plug-and-play’ instruments entering the market that automated the same principles to some extent. Importantly, however, since all these technologies depend on an antibody-based detection of proteins in a large variety of tissue types, the selection, validation and performance of the used antibodies have to be done with sufficient care. Adequate minimal validation guidelines need to be set, including the use of appropriate positive and negative controls (e.g. based on classical chromogenic stains (15)), to guarantee sensitivity, specificity and warrant the collection of biologically relevant data. Indeed, the performance of an antibody can vary enormously depending on the tissue type, the experimental conditions in which the antibody is applied, and whether formalin-fixed paraffin embedded (FFPE) or frozen materials are used (16–18). As such, the development and optimization of suitable antibody panels for multiplexed IHC still requires a significant amount of time, although externally validated reagents to detect commonly used markers are becoming more routinely available, for instance thanks to large scale initiatives such as the Protein Atlas consortium (19) or NIH initiatives (20).

**Figure 1:**
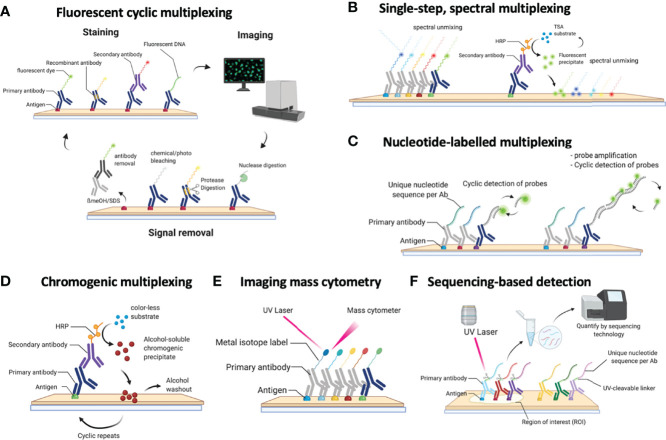
Schematic overview of the currently available methods for multiplexed immunohistochemistry (IHC). **(A)** Currently, the most common approach for multiplexed IHC makes use of fluorescently labelled probes, which are either directly coupled to the primary antibody or indirectly provided by a secondary antibody, that are detected in a cyclic fashion consisting of a staining protocol, followed by tissue imaging and signal removal. **(B)** In contrast to cyclic methods, single-step spectral methods detect all dyes in the tissue simultaneously: these can either be provided by directly labelled antibodies that are all simultaneously present in the tissue section or by the cyclic generation of TSA precipitates which are subsequently spectrally unmixed in a single imaging step. **(C)** Antibodies can also be detected by covalently linked nucleotide labels to which fluorescently labelled probes are hybridized in a cyclic fashion for which each cycle gets imaged. **(D)** Non-fluorescent mIHC methods involve the cyclic generation of chromogenic substrates that are washed away following an imaging step in between each cycle. **(E)** For imaging mass cytometry (IMC), antibodies are labelled with metal isotopes which are detected by the local vaporization of the metal ions by a UV laser, following which the present isotopes are resolved using atomic spectrometry. **(F)** Finally, nucleotide labelled antibodies can be detected by removing the nucleotide labels from the antibodies using a laser beam, following which the nucleotides that were collected from a precise region of interest are sequenced to quantify the amount of available proteins in that region.

The first step, even before exploring different multiplex staining methods, is figuring out if and/or how spatial analysis can help in answering your biological question. Spatial analysis can provide additional information on proximity-based cell-cell interactions, such as the infiltration of immune cells in a tissue, their proximity to tumor cells (see below). Subsequently, once it has been decided that spatial analysis will be needed to answer a particular biological question, the selection of the method becomes key (**Figure 1**, **Table 1**). As indicated above, the selection of the most appropriate method is largely defined by several parameters. First, the type (i.e. frozen *vs* FFPE) and size (i.e. needle biopsy *vs* tissue microarray (TMA) *vs* whole tissue slide) of a tissue, in addition to the number of samples that need to be analyzed (from 1 sample at the time to cohorts of hundreds of patients), will already define the first selection. As we will describe below, not all methods are compatible with FFPE/Frozen or large tissue samples, or can be easily scaled up to analyse hundreds of slides in a practical timeframe and with minimal variance induced by batch-effects. A second important parameter is linked to the number of markers that needs to be interrogated, a feature that is commonly highly project specific (e.g. 5 *vs* 50 markers). Finally, understanding how the currently available methods for multiplexed IHC work will be crucial to select the most appropriate one. (**Figure 1**, **Table 1**)

**Table 1:** Technical overview of multiplex IHC staining methodologies.

Method Name	Commercial Name	Sample	Max. n of Markers	Flexibility to implement new markers^¥^	Primary Ab	Ab tag	Signal removal technique	Tissue size	Automation	n of slides/experiment	Time (for 40-plex staining of 1 large slide)	Resolution	Tissue preservation	Current Scientific use^+^	Refs
MILAN	NA	FFPE	82	High	Unconj. and Conj.	FL sec ab	Ab stripping	WS/TMA	Partially automated	30+	2-3 weeks	subcellular	Yes	Mature	(21–23)
CycIF	NA	FFPE	60	High	Conj.	FL	bleaching	WS/TMA	Partially automated	30+	2-3 weeks	subcellular	Yes	Mature	(24)
MICS	MacSima™	FFPE/Frozen	400	Limited	Conj.	FL	bleaching/ fluorochrome release	WS/TMA	Fully automated	2	<1 week	subcellular	No	Limited	(25)
seqIF™	COMET™/ Labsat™	FFPE	40	High	Unconj. and Conj.	FL sec ab	Ab stripping	TMA/up to 2.2x2.2cm section	fully automated	1-4	1-2 days	subcellular	Yes	Limited	(26)
Chipcytometry	CellScape™ / ZellScanner ONE™	Frozen (FFPE not recommended)	65	High	Conj.	FL	bleaching	TMA/2x1 cm section	Service/Fully automated	NA	<1 week	subcellular	Yes	Mature	(27)
IMC™	Hyperion	FFPE/Frozen/Liquid biopsies	37	Limited	Conj.	Metal	None	WS+ROI TMA/slow	Service/automated detection	1	2 weeks	subcellular, 1 µm/px	No	Mature	(28)
MIBI™	IONpath/ MIBIscope™	Frozen	40+	Limited	Conj.	Metal	None	TMA/slow (ROIs 800 x 800 µm²)	automated detection	1	2 weeks	subcellular, 650 nm/px	No	Mature	(29)
NA	CODEX®	Frozen/FFPE	40+	Limited	Conj.	NUC	None	WS/TMA	automated detection	1	<1 week	subcellular	No	Mature	(30–32)
DSP	NanoString’s GeoMx® Digital Spatial Profiler (DSP)	FFPE	96	Limited	Conj.	NUC	None	ROI	automated, sequencing still required	1	<1 day	Subcellular/ grid	No	Mature	(33)
OPAL	Akoya’s Opal™ Multiplex IHC +/- Vectra® Polaris™ spectral scanner	FFPE	9	High	Conj.	HRP-TSA	Heat based Ab stripping (ER)	WS/TMA	Partially automated	30+	<1 day (for 9-plex)	subcellular	No	Mature	(34)
MxIF	Cell Dive (Leica)	FFPE	61	High	Conj. and unconj.	FL	Chemical inactivation	WS/TMA	Partially automated	30+	2-3 weeks	subcellular	Yes	Method published	(35)
cmIF	NA	FFPE	60	High	Conj.	FL	Chemical inactivation	WS/TMA	Partially automated	30+	2-3 weeks	subcellular	Yes		(36)
MELC	NA	Frozen	50+	High	Conj.	FL	bleaching	WS/TMA	Partially automated	1	2-3 weeks	subcellular	Yes	Mature	(37)
IBEX	NA	Frozen/FFPE	65+	High	Conj.	FL	Chemical inactivation	WS/TMA	Partially automated	30+	1-2 weeks	subcellular	Yes	Method published	(38)
NA	Orion RareCyte® platform	Frozen/FFPE	21	High	Conj.	FL	None	WS/TMA	Fully automated	NR	<1 day (for 6-plex)	subcellular	No	Method published	(39)
SABER	ImmunoSABER	FFPE/Frozen/Liquid biopsies	10	Limited	Conj.	NUC	None	WS/TMA	Partially automated	1	<1 day (for 10-plex)	subcellular	No	Method published	(40)
Ultivue	NA	FFPE/Frozen	8	Limited	Conj.	NUC	None	WS/TMA	partially automated	30+	<1 day (for 10-plex)	subcellular	Yes	Mature	(41)
SIMPLE	NA	FFPE	5+	High	Unconj.	HRP	Chemical Ab stripping	WS/TMA	Partially automated	30+	<1 day (for 5-plex)	subcellular	Yes	Method published	(42)
MICSSS	NA	FFPE	10	High	Unconj.	HRP	Chemical destaining + Ab blocking	WS/TMA	Partially automated	30+	<1 week (for 10-plex)	subcellular	Yes	Method published	(43–44)

NA, Not applicable; WS, whole slide; TMA, tissue microarray; FL, fluorescent; FFPE, formalin fixed paraffin embedded; Conj., conjugated; Ab, antibody; ROI, region of interest.

Overall, technologies for multiplexed IHC can be classified by the way the antibodies are administered and detected. Indeed, depending on the technology, large mixtures of antibodies (+20) can be administered simultaneously to a slide, following which a dedicated instrument is able to image, resolve and unmix the location of each antibody in the tissue. Alternatively, various methods also use cyclic procedures through which smaller amounts of antibodies (2–4) are used for staining and imaging, following which the detected signal is removed. By repeating this cycle multiple times, large numbers of markers can be detected in the same tissue section. Finally, several hybrid methods are available that combine both approaches or even use other technologies (e.g. NGS analysis) to resolve the complex mixtures of markers. In the first part of the review, we provide an extended overview of several of the currently available technologies for multiplexed IHC analysis on tissue sections, including their compatibility with materials, antibodies, throughput, plex level, timing, and compatibility with standard or dedicated instrumentation. Methods are grouped by the type of modification that is used to detect the various antibodies.

### Fluorescence-Based Detection of Antibody Mixtures

The largest group of methods for multiplexed IHC depends on the detection of mixtures of antibodies using fluorescent signals. Indeed, because fluorophores harbor specific excitation and emission spectra, they can be resolved using commonly used or more advanced microscopy tools, depending on how many fluorophores require resolving and their spectral overlap. In addition, most imaging instruments achieve a resolution below 0.6µm and are therefore perfectly compatible with single cell measurements [the average human nucleus is approximately 10 micrometers in diameter (45)]. A common issue with these methods is however the presence of autofluorescence in tissue: upon excitation with specific wavelengths, naturally occurring substances in tissue [for example extracellular matrix components, lipo-pigments, aromatic amino acids and flavins (46)] will emit light which overlaps with the fluorescence of the measured fluorophore, and needs to be considered and dealt with while processing images. Some chemical treatments (e.g. using bleach, sudan black or borohydrate) have been suggested to remove autofluorescence, but need to be used carefully and typically only solve the problem partially, while computational methods often offer better solutions, although in that case sufficient control images that capture autofluorescence need to be collected.

#### Cyclic Methods Using Fluorescent Antibody Detection

These methods make use of a cyclic procedure in which several steps (**Figure 1A**), including (i) the staining of small numbers of antibodies (2 to 4; typically defined by the microscope settings), (ii) the imaging of the sample and (iii) the removal of the stain, are repeated multiple times until all markers are detected. While cyclic methods are in general more time consuming, this approach allows to perform *interim* evaluations (allowing the researcher to validate every individual step and if needed repeat them), refine the composition of the panel during the procedure and adapt to unforeseen problems/results since cyclic methods conserve the tissue during analysis.

In these procedures, either directly labelled primary antibodies or labelled secondary antibodies are used to detect the markers. The former approach is used in the multi-epitope-ligand cartography (MELC) (37), multiplexed immunofluorescence (MxIF) (35), cyclic immunofluorescence (CyCIF) (24), Cyclic Multiplexed-Immunofluorescence (cmIF) (36), MACSima Imaging Cyclic staining (MICS) (25) and Iterative Bleaching Extends multi-pleXity (IBEX) (38) procedures. Indeed, MELC, MxIF, CmIF, IBEX and CyCIF make use of directly labelled antibodies which are usually combined in a triple/quadruple staining using 3/4 distinct fluorophores with non-overlapping spectra. Following staining and imaging, the fluorescent signal is removed using a photo-induced or chemical bleaching step before probing for the next markers (24, 35–38). This is different for the MICS technology, which makes use of recombinant antibodies from which the fluorescent label can be removed using a proprietary enzymatic cleavage reaction (25). In either case, antibodies [or the Fab fragments which are left after cleavage (20)] remain in the tissue when the next round of markers are added to the tissue slide. The latter is different in the MILAN approach (21–23), which makes use of fluorescently labelled secondary antibodies that bind to the specific primary antibodies. This has the advantage that signals can be amplified making it easier to detect weakly expressed markers, which might be an issue when using directly labeled antibodies without amplification. Another difference is that in the MILAN procedure, antibodies are entirely removed using an SDS/ßMercaptoethanol washing step to denature, inactivate, and remove the antibodies from the tissue (15), avoiding potential issues with antibody crowding or steric hindrance which could theoretically arise in methods that do not remove the antibodies (even though there is no formal evidence for such issues at the moment). The downside, however, is that the usage of secondary antibodies in MILAN forces users to make combinations of primary antibodies that were raised in different hosts or harbor different isotypes to avoid cross-reactivity during primary antibody detection (e.g. combinations of Mouse IgG1, Mouse IgG2, rat, goat and/or rabbit need to be made). On the other hand, directly labelled procedures depend on the direct labelling of antibodies, which requires careful selection of color combinations and carrier-free formulations of the primary antibody solutions (which may require custom made formulations), but, once available and validated, can be combined independent of the species where the antibody was raised.

In either of these methods, antibodies are commonly administered to the tissue in small batches (2 to 4) after which the tissue is imaged. Importantly, this setting allows the usage of regular microscopy and imaging tools using common fluorescent channels that are typically available in laboratories across the world. Moreover, automation of these procedures is gradually increasing, including the use of autostainers and automated slide scanners with regular fluorescent settings, and novel technologies using microfluidics (such as incorporated in the LABSAT and COMET system from Lunaphore, the CODEX system from Akoya (see below), or the MACSima instrument for MICS) are rendering it possible to further speed up the acquisition for small numbers of slides (26). The MICS method, on the other hand, has been optimized on a proprietary instrument, which allows unassisted, automatic processing of a small number of tissue sections (25). Similarly, chipcytometry (27) makes use of highly specialized equipment in which microchambers containing attached cells or (frozen) tissue can be mounted, and subsequently subjected to large multiplex analysis, which is primarily done in a cyclic way, one antibody at the time.

Finally, these methods are primarily compatible with FFPE tissue sections, while the MELC and MICS method have also been described to be compatible with frozen materials (15, 25, 47, 48). Overall, using these technologies, large numbers of markers can be analyzed (50+ have been reported (see **Table 1**), and the number of analytes keeps on rising constantly. Here, we added a new example of a melanoma tissue sample that was stained for 82 markers using the MILAN method (23) over the course of 50 rounds (**Supplement Figure 1**), while the MACSima technology was used to stain 327 markers in tonsil tissue over 160+ rounds (25). Overall, the primary limitation of the number of markers mainly comes from the compatibility of the tissue with the antibody removal procedure: methods involving chemical bleaching steps (such as in CyCIF and MxIF) eventually lead to tissue destruction, loss of antigenicity and/or tissue loss, and are therefore limited in the number of cycles that can be performed (47, 49). Also, cover slips that have to be added/removed repeatedly between the staining/imaging cycles, may cause tissue damage. Finally, the tissue type and how it was preserved prior to the start of the mIHC largely determines the plex level that can be achieved and needs to be defined experimentally.

Next to tissue damage, also the scalability of methods needs attention: while some methods (e.g. MELC, CyCIF and MILAN) allow the simultaneous processing of multiple slides, others (e.g. MICS, COMET) are limited to 2-4 slides at the time, although robotic systems that allow automated slide loading are in development. Also, the area that can be imaged/scanned can differ greatly among systems (from a few mm (2) to whole slide). Scaling can be further enhanced by compiling tissue microarrays (TMA), in which carefully selected tissue subsamples (also referred to as ‘cores’) from large tissue blocks are assembled on a single slide (typically 60 cores of 2mm diameter can be put on the same slide). Considering there is more heterogeneity observed across the dimensions of a single section than between different sections in tumors (50), one should deliberately design the TMA to capture this potential heterogeneity, by taking a smaller number of cores from small and homogeneous samples and a larger number in big and heterogeneous specimens. The role of the pathologist in selecting the different regions of interest and design of the TMA remains key. In this manner, it has been shown that different tumor biomarkers are accurately reported through the assessment of TMAs (51–53). As such, cohorts of 50-150 patients can be analyzed within the timeframe of a couple of weeks. This workflow is highly compatible within the research context, for instance in performing retrospective analyses simultaneously on big patient groups avoiding batch effects. However, within the daily clinical routine, implementation of TMAs in the workflow is less obvious, even though there may be a role for them when scan areas of more automated (faster) mIHC systems are too small to cover the entire tissue section as a whole and the analysis of multiple regions of interest is still needed.

#### Batch Methods Using Fluorescent Antibody Detection

In addition to cyclic procedures, novel methods are appearing that allow more extended, single-step multiplexing (**Figure 1B**). This is currently achieved by labelling antibodies with specific fluorophores which can subsequently be resolved using spectral unmixing methods, such as used in the RareCyte Orion system (39). The advantage of this approach is that a maximum of currently 21 antibodies are applied simultaneously in a single staining procedure, as such offering a fast staining of the sample. However, the generation of antibody panels containing 20+ different fluorescent dyes can be challenging, and requires dedicated instrumentation for spectral unmixing. Moreover, a significant amount of time (i.e. multiple hours) is required to collect high-resolution images from a small number of whole slides for each included channel. Finally, the simultaneous addition of all markers limits the flexibility to modify the panel after it has been validated, and the generation of the labelled antibodies remains cumbersome and requires a careful selection and validation of the multicolor panel. On the other hand, for routine purposes where the same panel has to be applied repeatedly, this method may offer excellent options, although this has not been investigated yet. Another recent approach enabling higher level multiplexing involves the UltraPlex, hapten-based technology, which adds labels to primary antibodies, which are subsequently detected by anti-hapten antibodies coupled to various fluorophores. While so far only lower plex panels (4-8-plex) were tested, it bears the potential to scale to routine assays of 12 or more markers (54).

#### Hybrid Approaches Using Fluorescent Marker Detection

In addition to cyclic and batch procedures, other methods use a hybrid approach to achieve extended multiplexing (**Figure 1C**). This is currently achieved by labelling antibodies with unique nucleotide barcodes which are subsequently detected using either direct hybridization of fluorescently labelled complementary nucleotide probes (as in the CODEX system (30–32)), or by using an *in-situ* amplification system (as used in the immunoSABER system (40) or the InSituPlex system (Ultivue) (41)). The hybrid nature of these approaches comes from the batch application of all antibodies simultaneously in a first step, while their subsequent detection using the detector probes is subsequently done in a cyclic fashion where hybridization/denaturation steps are alternated with imaging, with the latter needing to be repeated for each cycle (as such leading to a time scale comparable to the cyclic methods). An alternative approach was recently described in the SeqStain procedure (55), where antibodies labelled with fluorescently-labelled DNA (either primary or secondary) are used to detect protein markers, following which the DNA is removed by an enzymatic reaction using a nuclease. By repeating these cycles, multiple markers could be visualized.

These approaches require that antibodies are labelled with nucleotide probes, which reduces flexibility and speed to design novel panels. Moreover, the nucleotide sequence composition of the probes still requires significant optimization to avoid nonspecific binding. On the other hand, unlimited numbers of antibodies could theoretically be labelled with unique barcodes, and as such be combined in large quantities. It remains to be seen, however, how feasible such extended approach will be, whether and when steric hindrance/overcrowding will become an issue, and how destructive multiple rounds of hybridization/denaturation will be for the tissue structure, a step that will probably define the plex level. The cyclic application of the probes also requires specific instrumentation containing microfluidic devices and temperature control or repeated manual work, currently complicating the scalability of this approach to large batches of slides. Finally, while the CODEX was initially optimized for frozen materials, both methods are now compatible with either FFPE or frozen tissue sections (32, 40).

#### Detection Using Fluorescent Precipitates

Finally, while all the above-mentioned technologies allow the measurements of large numbers of protein markers, in many cases, researchers don’t need such complicated systems. Alternatively, a limited number of markers can be detected by using fluorescent precipitates (**Figure 1D**), for example by using tyramide signal amplifications (TSA) reagents, an enzyme-mediated detection method (34). The detection of antibodies in this system is based on a cyclic procedure, where each primary antibody is stained separately, probed with a Horse Radish peroxidase (HRP) secondary antibody, and then a specific fluorescent precipitate of the TSA reagent is generated. At the end of each cycle, the primary and secondary antibodies are removed while leaving the fluorescent precipitate before probing with the next antibody. Because all precipitates harbor a different fluorescent spectrum, imaging is only done once at the end, after which antibodies are spectrally unmixed. This concept is used in for example the OPAL system (34), allowing to perform 6-plex staining of FFPE tissue in an automated fashion using commonly available autostainers (typically present in pathology labs) and a dedicated spectral scanner. This technology is compatible with FFPE materials, and tens of slides can be stained simultaneously depending on the autostainer instruments used. In addition, it has to be said that some fluorescence-based detection systems are using directly linked primary antibodies. Therefore, it could be suggested that sensitivity is lower compared to the generally used chromogenic staining, which is often used with an amplification (frequently a polymer with several HRP enzymes) of the signal (56). Currently, there is a lack of good comparative studies which investigate this question. Some studies even suggest a comparable sensitivity (57, 58). A proper evaluation of the advantages and disadvantages of each technology regarding specific research/clinical question should be done.

### 2. Chromogenic Detection of Antibody Mixtures

Next to fluorescent detection methods, the use of more classical chromogens has been employed to develop methods for multiplexed IHC (**Figure 1E**). The Sequential immunoperoxidase labeling and erasing (SIMPLE (42)) or the multiplexed immunohistochemical consecutive staining on single slide (MICSSS (43, 44)), are methods that use the alcohol-soluble peroxidase substrate 3-amino-9-ethylcarbazole, a reagent that in the presence of the commonly used HRP and H_2_O_2_, generates a chromogenic, red precipitate that can be imaged using regular white light, brightfield microscopy. Once the image is collected, the red precipitate is washed out using ethanol following which the antibody is eluted using acidified permanganate. As such, 5 to 10-plex stains have been described (42, 43). While this method is largely compatible with standard equipment and procedures in pathology laboratories, it remains to be seen whether the tissue can withstand increased rounds of the low pH antibody washout buffer. Another recent approach using chromogenic multiplexing involves the UltraPlex, hapten-based technology, allowing to generate low-plex analyses (54). So far mainly FFPE tissues were processed, while frozen samples were not analyzed yet. Finally, while easy to assess whether markers are highly expressed or completely absent, the quantification of chromogens is far less quantitative than fluorescent dyes.

### 3. Mass-Cytometry Based Detection of Antibody Mixtures

As an alternative for chromophore/fluorescence-based imaging, imaging mass cytometry (IMC) makes use of metal-labelled antibodies, which are resolved using mass-spectrometry, an approach that is currently used in the Hyperion (28) or the multiplexed ion beam imaging (MIBI/IONPath) (29) systems (**Figure 1F**). At the moment, 42 purified metal isotopes, mostly from the lanthanide series, are commercially available for labelling purposes, although the theoretical amount could be 135 based on the possible isotopes, a number that is mostly limited by the excavation of these metal isotopes in sufficient amounts and purity. The analysis of tissues using IMC involves the staining of the tissue with the mixture of all pre-titrated antibodies together, following which laser-assisted ionization allows the analysis of the generated cloud containing the metal ions in a connected mass cytometer. The batch application of antibodies also makes it compatible with both frozen and FFPE materials. The resolution is currently 0.3-1µm, dependent on the system and settings, but imaging is rather slow at a rate of 2h/mm (2), which puts some constraints on the analysis of whole tissue slides or large cohorts of patients. Finally, similar to several other methods, labelling of antibodies is required, although commercial kits are available that are sufficiently easy to use (59).

### 4. Sequencing-Based Detection of Antibody Mixtures

A final approach to perform high-dimensional multiplexed IHC involves the combination of nucleotide-labelled antibodies and next-generation sequencing (NGS) (**Figure 1G**). Indeed, the Digital Spatial Profiling platform (DSP) makes use of photo-cleavable probes to label primary antibodies (33). By staining tissues with these antibodies, precise illumination of specific regions of interest in the tissue using a dedicated platform allows the isolation of the cleaved nucleotides which are subsequently quantified using NGS. As such, multiplexing of up to 40 proteins (or 5,000 mRNA probes) has been described (33), but this technology allows up to 800- of 80-plex profiling of either mRNA or protein, respectively, using an optical barcode readout and has the potential for even greater multiplexing using an NGS readout. This method is also compatible with both FFPE and frozen materials. However a minimum of (non-adjacent) cells (10-20 cells for protein, 50-200 cells for RNA (60)) is required to obtain sufficient probes to achieve high quality NGS data. As such, this technology does not yet achieve true single cell analysis, although specific cell populations could be profiled in depth. The selection of the cells to study, a critical step in this technology, can still be done by performing a lowplex IHC staining prior to probe isolation, but the isolation of specific, more complex, rare phenotypes could be hampered by this low-plex staining procedure.

## Data analysis tools for pathological interpretation

**Figure 2:**
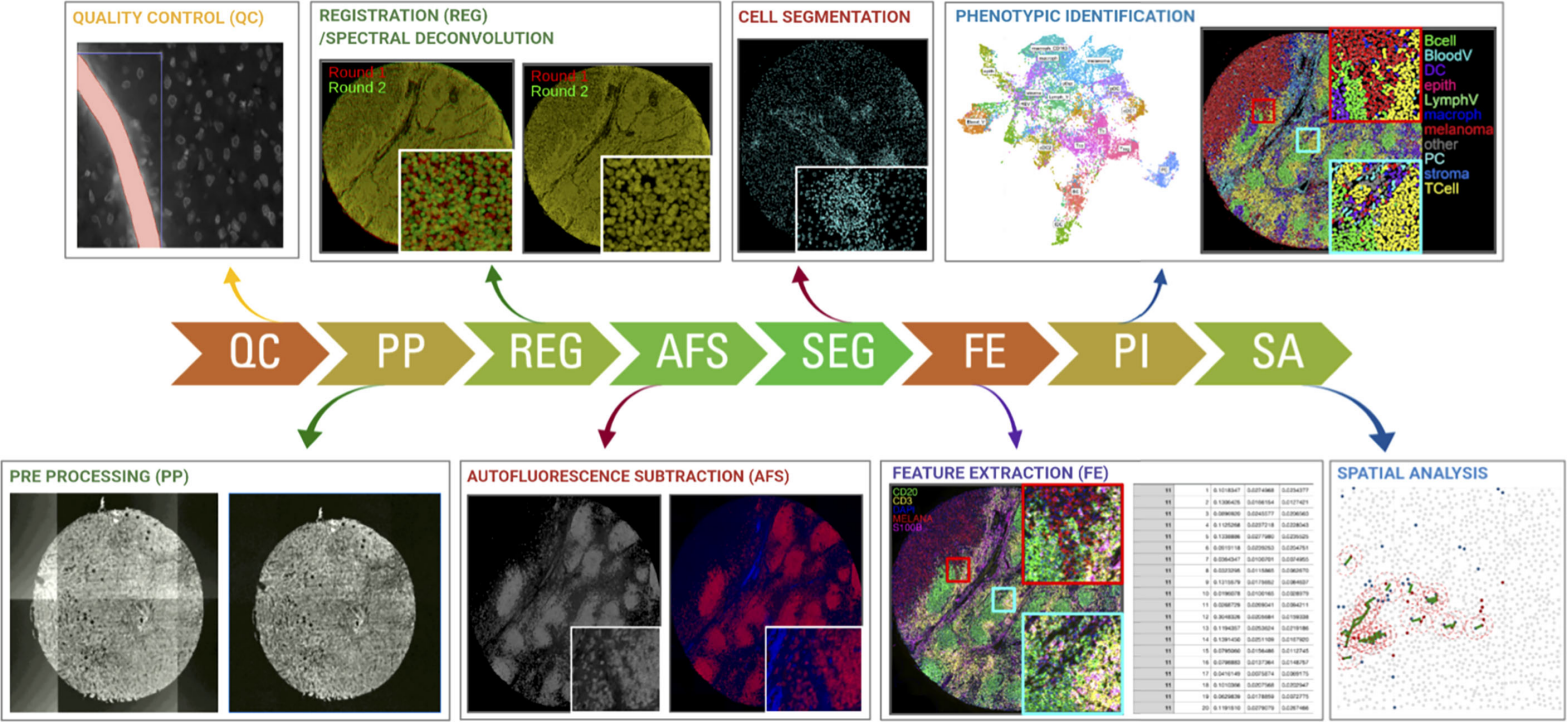
Schematic overview of the required steps for downstream image analysis using the most commonly used fluorescent, cyclic methods for multiplexed IHC. Images are collected across multiple cycles but still need to be cleaned (QC), corrected (PP), registered/aligned (REG), autofluorescence removed (AF), segmented (SEG), feature extracted (FE), phenotypically annotated (PI), and spatially resolved (SA).

As described above, a multitude of technologies are currently available to measure dozens of protein markers using multiplexed IHC at single-cell level in a tissue slide. Most technologies make use of automated slide scanners, which are able to image a wide range of tissue areas, from pre-selected (small) regions of interest (ROIs) to entire slides/tissue samples. Such output is subsequently subject to a detailed analysis process. Indeed, while the ‘wet lab’ procedures may still be adopted relatively quickly and easily by laboratories, the subsequent image analysis and interpretation of the resulting high-dimensional data still faces enormous challenges. In the second part, we therefore describe a general workflow of methods to perform data-analysis and extract the most relevant information (**Figure 2**). Also, while methods for imaging-based cellular analysis are well described (61) (e.g. as used in high content screening approaches of *in vitro* cultured cells), analysing images from tissue samples typically comes along with various additional challenges. Overall, analysing images from multiplexed IHC consists of 2 major steps, including (i) image-analysis and (ii) high-dimensional, spatially resolved data analysis. Image analysis refers to the extraction of quantitative and meaningful information from the image by means of digital processing techniques and can be further divided in two fractions: low-level and high-level processing. In low-level image processing, a digital image is used as input and another digital image is obtained as output (e.g., a corrected image for improved visualization/analysis), while high-level processing involves functions whose outcome is a description of the content of the input image such as cell edges or tissue regions. The reader should note that upstream steps such as image-acquisition fall outside the scope of this review and are not covered here.

### Low-Level Image Analysis

During low-level image analysis, all collected images are prepared for data extraction. While the procedures can be different between the various technology platforms, it generally shares a common strategy. Considering that the majority of technologies make use of fluorescent detection, we will primarily focus on these procedures.

When dealing with immunofluorescent images of tissue sections, these are often affected by aberrations which are critical to the quality of the results (62). Examples of these aberrations include out-of-focus regions (blurriness), vignetting effects, saturation debris, and artifacts due to e.g. air bubbles and tissue folds. Various methods have been described to tackle some of these aberrations. Examples include ConvFocus (63) from Google AI or FQPath (64) for blurriness detection (focus), CIDRE (65) that uses an energy minimization model and a flat-field correction method developed by Kask and colleagues which uses an additive and a multiplicative component to correct for field-of-view artifacts (vignetting) (66), a protocol implemented in CellProfiler (67) which uses supervised machine learning for automatic quality control of image-based measurements, and HistoQC (62) which implements an automated, quantifiable, quality control process for identifying artifacts and measuring slide quality.

On top of undesired impurities, IF images are also subject to other sources of fluorescent signals such as background and tissue autofluorescence that need to be tackled before quantifying true signals. Examples of methods addressing this issue include dark pixel intensity identification (68) which estimates background signals by acquiring images at a different set of exposure times, and an autofluorescence removal method that uses non-negative matrix factorization that separates the signal into true signal and autofluorescence components (69). Moreover, high-throughput experiments can be subject to batch effects, that is, part of the acquired signal is described by undesired technical variation (for example, sample manipulation) rather than biological sources (61). An example of a method to tackle this issue is RESTORE (70) which identifies negative control cells for each marker and uses their expression levels to normalize and remove sample-to-sample variation. Local/grid-based normalization tools (71) have also been developed, although their general implementation still requires a broader implementation.

In cyclic methods where images are acquired in consecutive rounds, the next step consists of the exact super-positioning of images acquired from consecutive imaging rounds (commonly referred to as ‘image registration (54)). Registration of medical images is a need that has been around for a while and thus a large number of methods are available. These methods can be broadly classified as rigid (image transformation is limited to translation, rotation, and scaling) (72, 73) and plastic (the moving image can be elastically deformed to best match the fixed image) (74). Historically, image registration was meeting a macroscopic need (for example, overlapping of MRI and Xray images). For multiplexing however, microscopic precision is required as a shift of a few pixels can overlap completely different cells. Therefore, recently, new registration methods have been published in the literature which aim to reach the required precision for exact-cell overlapping (75–78). While usually not required in “batch” methods (see above), this seemingly trivial step can be extremely computationally ‘expensive’ for large images (>100 million pixels) when applying plastic methods. Therefore, the latest algorithms come with GPU implementation. Also, depending on how images are acquired in the imager (e.g. some imagers scan all fluorescent channels at each step, while others scan the entire sample for each channel separately), additional registration of the acquired fluorescent channels may be required.

Image pre-processing requires a very systematic approach and should be robust and identical across all samples/images of the same project to avoid the loss of biological information and a potentially biased interpretation of the results. While computational tools exist tackling the individual issues, their implementation for non-expert users is challenging and requires some level of computational knowledge. Moreover, linking the inputs and outputs of consecutive steps is far from trivial and integrated workflows are still missing. All of this together makes that to date, image pre-processing is mainly done by manual/visual inspection in the routine setting, thus largely limiting the throughput of these techniques.

### High-Level Image Analysis

During high-level image analysis, the output of the different functions is a description of the content of the input image(s) such as cell edges, cell features or tissue regions. Once a set of images is properly aligned and ‘cleaned’, cells need to be precisely delineated (commonly described as ‘cell segmentation’). The quality of this step is very important for proper cell identification (see below). In tissue sections, this step is commonly done using nuclear segmentation. Historically nuclear segmentation has been tackled mechanistically (watershed algorithm and variations (79, 80). However, the introduction of deep learning algorithms (specifically convolutional neural networks (CNNs) like uNets) have outperformed all the mechanistic algorithms and now represent the state-of-the-art (81, 82). The main drawback of deep-learning based algorithms is the need for large training datasets. However, current efforts are ongoing to implement machine learning approaches (83), which are gradually becoming more efficient at recognizing and splitting cells requiring minimal hands-on image training (84, 85). While a few algorithms have explored the possibility of segmenting full cells (including cytoplasm and membrane) (86, 87) cell nuclei are far less heterogeneous making nuclear segmentation more robust. Regarding the clinical implementation of cell segmentation approaches, deep-learning based methods will require to fine-tune existing algorithms to optimize the accuracy of the predictions and adjust it to the specific acquisition instrument and sample material. To the best of our knowledge, such an ambitious comparison in immunofluorescent images and in a multi-center setting with a variety of acquisition instruments is not available in the literature. However, a similar study has been carried out for Ki-67 expression, a prognostic marker in breast cancer, which has shown excellent accuracy and reproducibility in different analysis platforms performed by multiple operators (88). Accuracy and reproducibility are well known issues in state-of-the-art histopathology where the sample readouts are extracted by the subjective eye-rolling of an expert pathologist which causes intra- and inter-observer variability (89, 90). In fact, the main limitation for the automation (human or computational) of pathological evaluations is the lack of standardization (91). However, we believe that with the right standardized roadmaps to histopathological analysis integrated computational pipelines will become state-of-the-art and that the future of pathology is mainly digital (92).

Finally, the acquired signals, such as fluorescence intensity, amount of metal isotopes, read-counts, etc., are quantified in every cell together with morphological features of the nucleus (nuclear size, shape, etc.) and topological features (X/Y coordinates) in a step called ‘feature extraction’, and collected in a structured data matrix.

Overall image pre-processing is highly dependent on the technology that was used to acquire the images – each of them will still require significant tweaks and adaptations to translate the procedures of one technology to another. While most vendors of the above described instruments provide accompanying software, these commonly don’t extend beyond mere viewing of images, a step that is crucial for quality assessments of the stains, but don’t allow quantitative and extended analysis. More dedicated (commercial) image analysis platforms are also gradually becoming available (e.g. Halo (Indica labs) or Visiopharm software packages for digital pathology, and the open source tools such as QuPath (93), CellProfiler (94), or HistoCat (95), are continuously updated, but considering the enormous amounts of biological questions that still require downstream analysis of the spatially resolved cell types, such packages typically remain constricted to initial groundwork, or highly dedicated to one particular type of analysis. Some more integrative pipelines are also gradually being released, such as MCMIRCO (96) or SIMPLI (97), although these still require a high level of bioinformatics skills.

### High-Dimensional, Spatially Resolved Data Analysis

Next, the obtained data matrix containing the high-dimensional data still requires further analysis and interpretation. A first step in this process involves the identification of the various cellular (pheno)types (i.e. epithelial cells, particular T cell subtypes, tumor cells, blood vessels, etc), a step that is usually done using clustering analysis and manual interpretation according to methods that resemble many other single cell methodologies. More automated algorithms that can be trained to assign labels to each cell (e.g. using convolutional neural networks (43)), and pretrained templates (98) do exist, but still require significant training, as cellular phenotypes can vary extensively between organs and/or disease conditions. In addition, this step is commonly further complicated by imperfect cell segmentation, as markers of adjacent cells can “pollute” neighboring cells, making interpretation difficult. This can require an interactive iteration of the settings for cell segmentation, next to a step-wise approach in clustering, where in a first step the major cell types are defined, after which the different subtypes of these cells can be defined. The latter is mainly used to avoid that large populations outcompete small/rare populations.

Regarding clustering, there are several methods publicly available without any single method proving to perform better than the others. While many studies have used one or another clustering algorithm (49, 99–101), our group implemented a *consensus* clustering procedure (31, 35) following a “Wisdom of the crowds” type of approach, where three independent algorithms (to choose between Phenograph, K-means, FlowSom, ClusterX, Clara, Hierarchical clustering, among various others) were used to cluster the identified cells. Each of the identified clusters is then annotated manually by an expert pathologist/immunologist, and only cells that remain in the same annotated cell type across minimal 2 methods are retained for further analysis. Since multiplexing datasets can identify millions of cells, clustering is done in a subset of these cells. There are different sampling strategies available, including random sampling, stratified random sampling, stratified proportional random sampling, etc. The annotated subset is then projected on the entire dataset using cell-type-specific fingerprints. The above-described methodology, however, still requires significant manual work from expert pathologists/immunologists, a step that could still largely benefit from properly curated databases, which will allow for machine learning tools to automatically recognize and annotate cell types.

Once this information has been gathered, data is subsequently projected against the spatial context of the tissue. Indeed, each cell is composed of a precise subset of pixels with specific coordinates that span a specific surface in the image. This information therefore allows the analysis the spatial distribution of the cells that can be used to model cell-cell interactions (32) (typically referred to as ‘neighborhood analysis’) and any other spatial measurement (e.g. distance measurements to structures of interest, nearest-neighbor analysis, etc), but also the definition of larger cell communities and tissue architectures (102). For any of these analysis subtypes, in spite of the presence of several methods implemented in papers published in literature (102–106), there are currently very few software packages available, mostly requiring specific and specialized bioinformatics skills. However, from a biological point of view, this step is the most crucial as it will allow researchers to explore complex biological systems in a quantitative way, so sufficient efforts will still be required in the coming years to develop standardized methods. Moreover, the high complexity of these analyses, together with the relatively small scanned areas or amount of slides/patients (which cannot capture the full heterogeneity of a tissue or disease group), typically results in large patient-to-patient variation, which will require that significantly large cohorts are interrogated to come to statistically robust conclusions. Whether sufficiently large cohorts can be analyzed will in great part be defined by the used platform (see above). Also, diagnostic tools using mIHC/IF have shown their benefits for the treatment of the patient. Standardized methods to perform image analysis will have to be developed and properly validated before their clinical implementation can even be considered.

Finally, each described method will eventually generate terabytes of images containing expression profiles of each included marker. The size of such data sets poses an additional challenge to store, label, transfer, computationally process and interpret the data in a reasonable amount of time. Current methods for big data processing, however, should be easy to adopt, but are only now becoming available in most institutes. In line with this, over the coming years, this “next-generation” pathology field will also need extended digital data repositories and standardized analysis methods to bring diagnostics and tissue interpretation to the next level. Implementing the required safety measures in line with the current General Data Protection Regulation (GDPR) regulations will also be of primary importance (107).

## Making the difference with spatial IHC profiling

In the last part of this review, we provide an overview on how multiplexed IHC has been used and implemented in research over the past years. Considering the growing number of papers (all to which we can unfortunately not refer), we chose to focus on the most important concepts and provide examples of how researchers have used (some) of the above described methods together with dedicated bioinformatics analysis pipelines to gain additional insights in complex biological processes.

### The General use of Multiplexed IHC Across Research

**Figure 3:**
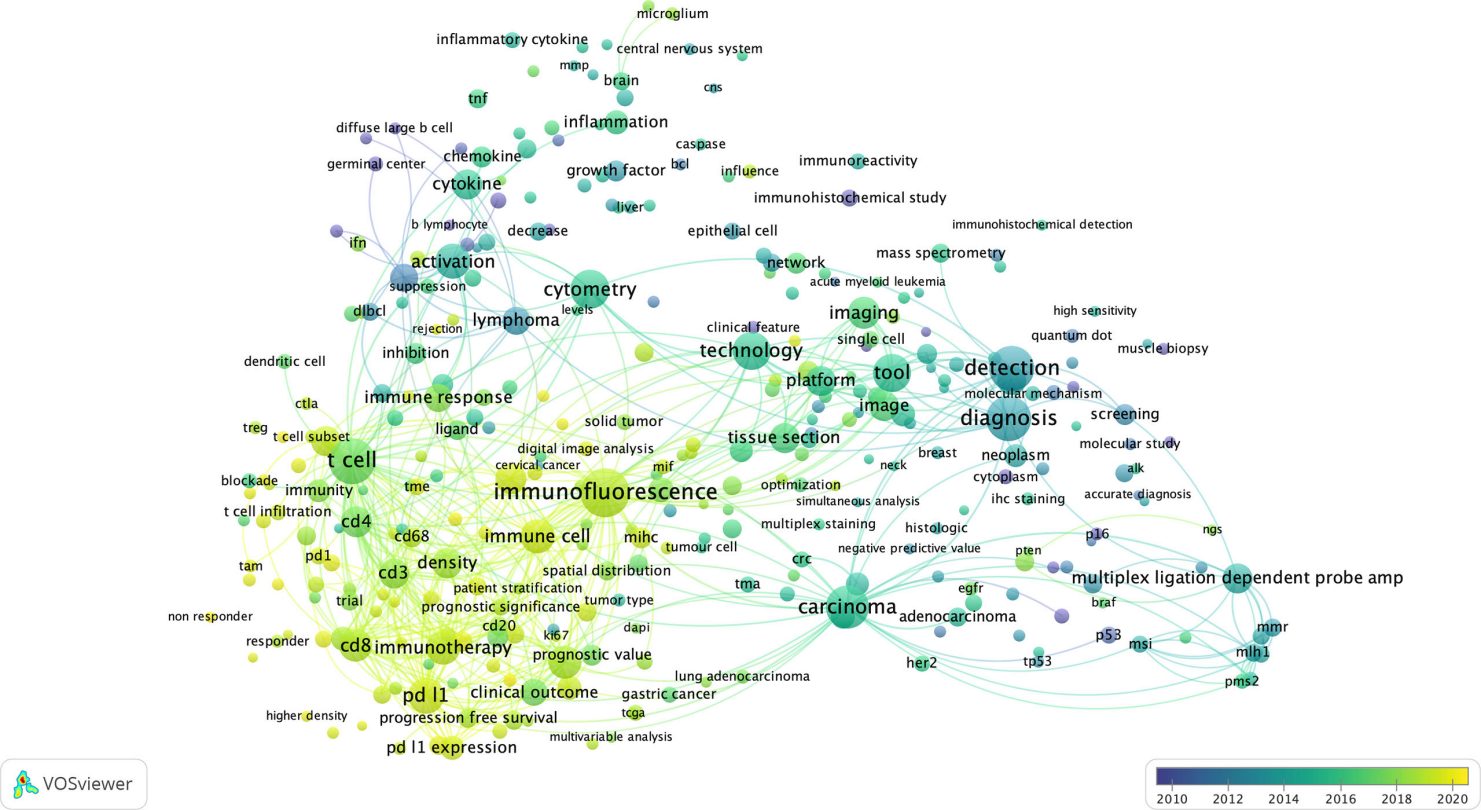
The bibliometric map of multiplexed IHC over the years. Using VOS Viewer, a software tool for constructing and visualizing the bibliometric network related to “multiplexed immunohistochemistry” on PUBMED (see *Methods*), we observed a shift from 2010 where technology development started (blue circles), to its use to unravel complex cellular networks in 2020 (yellow areas) with a strong focus on immuno-oncology and T-cell biology.

First, to get a top-level view on how multiplexed IHC has been used over the past years, we have performed an in-depth literature search, for which keywords and co-occurring terms were extracted from ~1000 papers from PUBMED using a query related to “multiplexed immunohistochemistry” (see *Methods*), and generated scientific networks. Based on the most occurring terms in the titles and abstracts of these papers, we can draw two main conclusions (**Figure 3**):

First, considering the topics of research, we identified 3 main topics: (i) technology/methodology development for multiplexed IHC using antibodies in tissue sections with the majority of methods focused on the usage of immunofluorescence; (ii) a strong focus on cancer research, with the aim to better predict prognosis, outcome and survival of patients based on the analysis of tissue sections; (iii) the vast majority of research papers used multiplexed IHC for the analysis of immune infiltrates, with a primary focus on T cell biology, checkpoint inhibition, macrophages and B cells. In addition, a more detailed density map also shows several prominent biological markers (e.g. CD3, CD4, CD8, CD68, CD163, CD20, PD1/PDL1, CTLA4, etc) that are generally used for T cell profiling, checkpoint analysis, and the investigation of macrophages and B-cells.

Second, when overlaying the generated network with the dates of when the papers containing the indicated terms were published, we identify a trend where research was initially focused on technology development (before 2016), while gradually moving to its implementation in the research community to study immune infiltrates related to cancer development and its treatment using immunotherapy (2016-2020).

### Using multiplexed IHC to answer complex biological questions

Considering the rise in available technologies for multiplexed IHC over the past few years and their gradual implementation, only the tip of the iceberg has been uncovered. Indeed, next to the revolution in single cell profiling, for which single-cell RNA (scRNA) sequencing is still the primary method for cellular phenotype exploration (108), the implementation of spatial technologies is becoming increasingly important to precisely locate the identified cell types and phenotypes in the context of a tissue and across a patient cohort. In this way, accurate atlases of healthy and disease-specific microenvironments can be compiled, both for human and mouse tissues (109, 110). For instance, the myeloid compartment of glioblastoma brain tumors was recently described using the combination of scRNAseq, CITEseq and multiplexed IHC through which the various ontologies of the infiltrating macrophages and resident microglial cells could be identified, while their distributions were spatially separated over various niches, which, moreover, evolved from the newly diagnosed to the recurrent setting (111). The distribution of the various cells across a tissue section was determined by computationally breaking up the tissue in smaller “tiles” in which the relative distributions of the identified cell types were determined. The same approach is directly applicable to define tumoral, peritumoral, perivascular and non-tumoral areas (112), and, while these insights are key to define the macrostructure of a tissue, such analysis still remains on the level of one cell type at the time. Once macro-level areas are defined, it becomes key to understand local cellular distributions, an analysis type that is done by performing neighborhood or nearest neighbor analyses. In this analysis, the local neighborhood from each individual cell is defined by assessing which other cell types are present within a certain radius or distance. We and others have used this approach to define local cell-cell interactions between all identified immune cell populations in melanoma samples (23, 96). As such, it was found that exhausted cytotoxic CD8+ T cells were typically residing in close vicinity to TIM3- or PDL1-positive macrophages (23, 113, 114). Similarly, it was found that the CD8+/TCF7+ double positive T cells that were residing in the tumor could be reactivated and showed a positive correlation to responsiveness to checkpoint blockers in melanoma (115).

Such inference could only be achieved by performing multiplex analysis in tissue sections, allowing researchers to simultaneously define the phenotypic and functional status of each identified cell type but also define the spatial distribution of each cell and how they relate to each other. A similar cellular sociology was identified in breast tumors using IMC through which *communities* and local niches could be determined which typically consisted of a dominant tumor clone in combination with a variety of immune cells (102). Cellular neighborhoods could also be identified in colorectal tumors (116). Moreover, by measuring such neighborhoods, the presence of particular CD4+ T cells in cellular neighborhoods that were also enriched for granulocytes was identified as a positive markers for survival in CRC, while the presence of macrophages in breast tumor communities rather correlated with poor prognosis (102, 116). Also in lung, ovarian, liver, kidney and various other cancer types, the presence of specific immune cell infiltrates were shown to correlate with good or poor outcomes (117–119) or with response to immunotherapy (120). The number of research papers using this type of analysis keeps on rising steeply (and we apologize to all the authors of papers we have not mentioned here), even though the scale at which such projects can be performed should become even larger to achieve the next clinical revolution.

## Multiplexed IHC vs. spatial transcriptomics: a complementary duo

In this review, we have mainly focused on methods for multiplexed IHC. The main advantage of these methods resides in their prompt translatability to the clinical setting: indeed, pathology labs have been performing antibody-based assessments for decades and adding mIHC should be more easily adoptable in such setting, even though a transition towards digital pathology will be required. Recently, methods for spatial transcriptomics are being developed at rapid pace: the main advantage of the latter methods (for which a plethora of technical approaches is currently available (121)) is their more universal character, whereby complementary detection probes can be developed in a more generic and species independent way, while mIHC largely depends on the availability of specific, high quality antibodies, which are not always available and often not cross-reactive over species (e.g. mouse vs human). While such approach enables researchers to uncover unknown pathways and patterns, RNA-based methods also harbor some pitfalls as well. Indeed, for the majority of (archival) samples, the currently available standard quality of materials (as they are mostly available as FFPE materials in biobanks across the world, and less often in the form of fresh frozen tissue blocks) is typically directly amenable for antibody-based approaches leading to robust insights. The quality of samples for RNA based analyses will, on the other hand, require very close monitoring of sample/RNA quality, as the stability and longevity of RNA molecules is much less compared to proteins. The translational character of RNA-based methods will therefore still require proper benchmarking to ensure robust pathology grade readouts. The coming months and years will have to show how this exciting field keeps on evolving.

Finally, both protein and RNA-based methods are gaining more and more traction to unravel complex cellular networks and architectures. However, the main setting currently still relies on the “discrete” utilization of one method at the time. Indeed, while each method can already provide highly valuable insights at single-cell resolution, each approach also harbors various downsides as well. For instance, while transcriptome analysis is excellent at unravelling transcription factor networks or identify the source of cytokine expression in complex tissues (122), signal-transduction events, protein-protein interactions or particular immune cell states can be measured more reliably at the protein level (123). As such, combining multiple methodologies in an orchestrated fashion can produce highly synergistic insights that cannot be achieved by either method alone. This evolution towards multi-omics approaches is a very active domain, where various challenges will have to be overcome as well. Not only will methods have to be adapted in such way that capturing RNA and protein based features will remain possible, also data integration will require further evolution, even though steps are being put in that direction (121, 122).

## Conclusion

A large variety of technologies for multiplexed immunohistochemistry has been developed over the past years, each with their own advantages and downsides, and it is expected that these will keep on improving over the coming years. Moreover, combinations with other spatial omics, such spatial transcriptomics, genomics, chromatin accessibility, lipidomics or metabolomics, are underway which bring yet another level of technological and computational challenges. Regardless of the approach, each technology has the goal to interrogate increasing numbers of analytes in pathological tissue samples at single-cell and spatial resolution. This revolution allows us now to investigate complex patho-biological processes at unseen resolution- insights that bear the potential of becoming the next generation of higher order biomarkers. This can, however, only be achieved if appropriate computational tools and infrastructure – the hallmark of a true shift towards digital pathology – are implemented that can deal with this approach and complexity. Moreover, considering the number of parameters that will be measured, it will be paramount to investigate sufficiently large populations of patients so that we can evolve from anecdotical case reports to more fundamental, robust and clinically useful insights. The latter will require the combination of highly standardized methodological processes and concurrent validated analysis pipelines. Finally, such next-generation pathology will also require standardized digital data repositories to set appropriate standards and benchmarks to bring the field to the next level.

## Methods

### Digital Literature Analysis

The VOSViewer tool and algorithms (Visualizing scientific landscapes; https://www.vosviewer.com (124);) were used to analyse extracted publication data from a PubMed search using the following search criteria:

(multiplex immunohistochemistry[Title/Abstract]) OR

(multiplexed immunohistochemistry[Title/Abstract]) OR

(multiplexed immunofluorescence[Title/Abstract]) OR

(multiplexed immunophenotyping[Title/Abstract]) OR

(multiplex immunofluorescence[Title/Abstract]) OR

(multiplex immunophenotyping[Title/Abstract])

### Multiplexed Analysis using MILAN

A 3µm thick tissue section of a melanoma tissue microarray was subjected to multiplexed immunohistochemistry according to the MILAN protocol as previously described (21). Overall 83 antibodies were succesfully used as have been described here (23, 125).

## Author Contributions

FS and FB conceptualized the manuscript. All authors contributed to the writing of the manuscript. All authors contributed to the article and approved the submitted version.

## Funding

This work was supported by KULeuven funding (C3/19/053, C1/17/084), the Opening the Future foundation, the Kom op tegen Kanker (Stand up to Cancer) foundation, FWO infrastructure grant (I005920N) and an FWO Fundamenteel Klinisch Mandaat (EMH-D8972-FKM/20) to FB.

## Conflict of Interest

The authors declare that the research was conducted in the absence of any commercial or financial relationships that could be construed as a potential conflict of interest.

## Publisher’s Note

All claims expressed in this article are solely those of the authors and do not necessarily represent those of their affiliated organizations, or those of the publisher, the editors and the reviewers. Any product that may be evaluated in this article, or claim that may be made by its manufacturer, is not guaranteed or endorsed by the publisher.

## Supplementary Material

Supplementary Figure 1 GIF representation covering an 82-plex of a melanoma tissue sample stained with 52 consecutive rounds of MILAN staining.
