# Supplementary figures and images for: Next-Generation Pathology Using Multiplexed Immunohistochemistry: Mapping Tissue Architecture at Single-Cell Level
Source: Front Oncol. 2022 Jul 29;12:918900. doi: 10.3389/fonc.2022.918900 (PMC9389457; doi:10.3389/fonc.2022.918900)

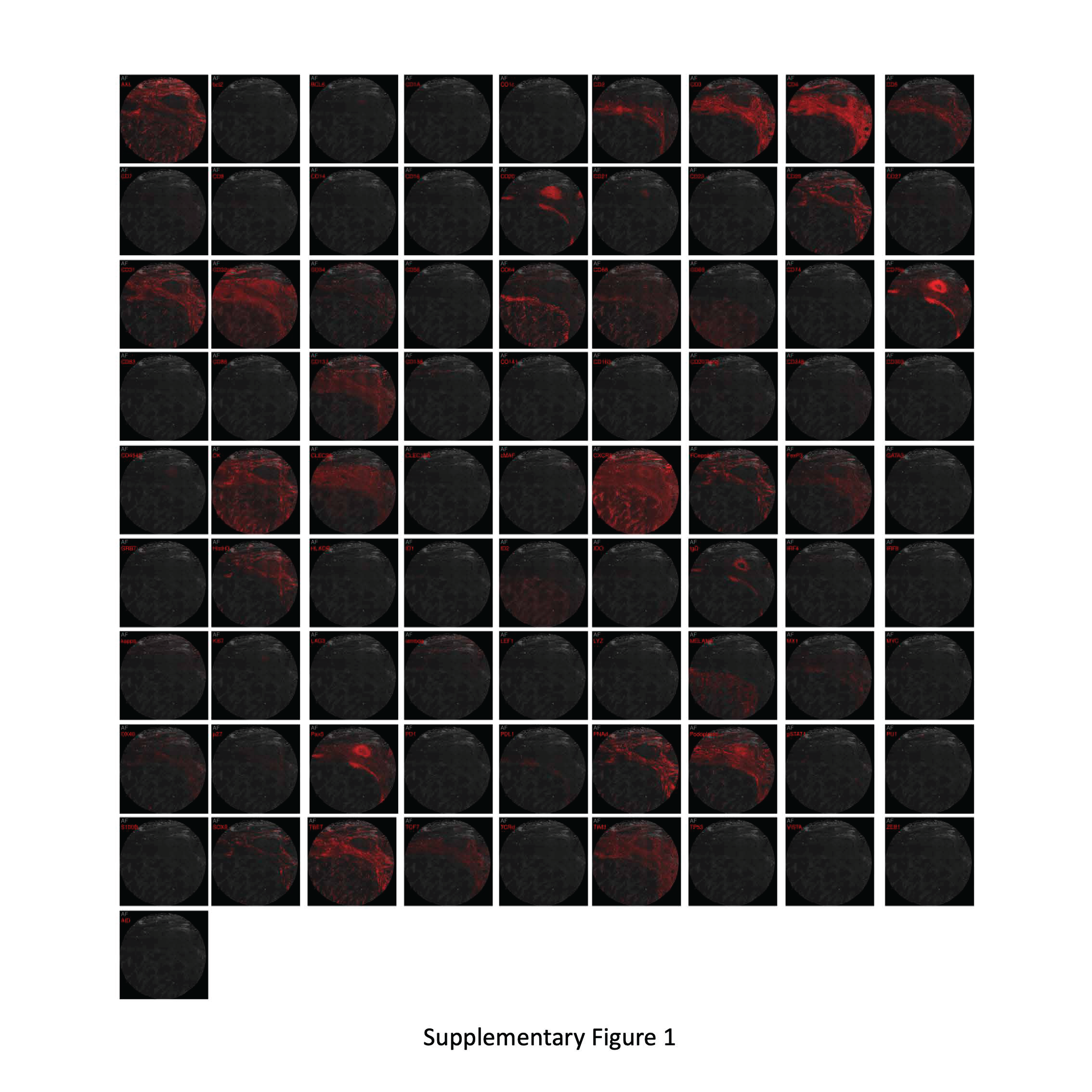

Supplement: Supplementary Figure 1 — GIF representation covering an 82-plex of a melanoma tissue sample stained with 52 consecutive rounds of MILAN staining. [file Image_1.tiff]
